# Modeling the microRNA regulation of TGF-*β*/SMAD signaling pathways for seizure control in temporal lobe epilepsy

**DOI:** 10.1038/s41540-025-00643-6

**Published:** 2026-01-15

**Authors:** Kurt J. A. Pumares, Daniel P. Martins, Aiman Khalil, Jochen H. M. Prehn, Deirdre Kilbane

**Affiliations:** 1 Walton Institute, South East Technological University, Waterford, Republic of Ireland; 2 School of Computer Science and Electronic Engineering, University of Essex, Colchester, UK; 3 Department of Physiology and Medical Physics, Royal College of Surgeons in Ireland, Dublin, Republic of Ireland

**Keywords:** Biochemical networks, Differential equations, Dynamical systems, Regulatory networks, Computational biology and bioinformatics, Neuroscience, Systems biology

## Abstract

Temporal lobe epilepsy (TLE) is the most prevalent type of focal epilepsy. Recent developments in sequencing, proteomics and network analysis tools provide new avenues for investigating potential molecular therapeutic targets. Both the TGF-*β*/SMAD signaling pathways and subsets of microRNAs (including miR-21a-5p, miR-142a-5p, and miR-10a-5p) have been shown to be altered in several preclinical models of epilepsy and were mathematically modeled in this study. Using prior systems-based findings, a changeover between ‘seizure’ and ‘anti-seizure’ cellular states has been identified upon inhibition of microRNA activity achieved by the injection of antagomirs. Methods for seizure suppression were explored under various antagomir dosages as well as the regulatory effect of each microRNA in order to ascertain intracellular responses. Promising antagomir administration strategies were then identified, which may offer new avenues for seizure suppression.

## Introduction

Epilepsy is the result of various temporary abnormalities in the brain function called seizures, which are generated by the hyper-excitability and hyper-synchrony of neuronal networks^1,2^. The International League Against Epilepsy categorizes seizures depending on the location of their onset in the brain, i.e., (1) *focal* when generated in restricted regions, (2) *generalized* when spread to the entire cerebral hemisphere, and (3) *unknown* when there is insufficient information available about their onset^3,4^. Clinical manifestations of epilepsy, such as irregular convulsions and altered consciousness, have major consequences on the daily functions of the affected patients, as well as their physical and mental health^5^. In addition, various other neurological diseases and conditions, such as tumors, infections, and strokes, may also induce epilepsy, making it the second most common life-threatening neurological emergency^6^.

Temporal lobe epilepsy (TLE) is the most common form of focal epilepsy in which seizures often begin in the hippocampus or its surrounding area^7^. The most common pathological findings are selective neuron loss and gliosis within the hippocampus of patients, which also features neuroinflammation and remodeling of neuronal networks^8–10^. It is estimated that 22.5% of epileptic patients have drug-resistant epilepsy (DRE), which therefore requires an early evaluation for surgical treatment (temporal lobe resection)^11^. Many antiepileptic drugs prevent seizures but might not appropriately target the underlying pathogenic processes and instead have side effects on neuronal excitation and inhibition^12^. Moreover, only less than 1% of traditional drugs can penetrate the blood–brain barrier (BBB), which serves as an anatomical and biochemical dynamic barrier in the brain protecting it from systemic circulating and externally injected molecules, unfortunately posing a key challenge for drug delivery into the brain^13–15^. Systems biology approaches produced new insights into the molecular pathophysiology with the advancements in sequencing and array-based profiling of protein-coding and non-coding transcripts, thereby identifying new molecules for therapeutic targeting in epilepsy^16–19^.

Spatial and temporal changes in microRNA (miRNA) expressions were found when *status epilepticus* (SE) is used to trigger epileptogenesis, notably in the hippocampus^20–25^. miRNAs are endogenous small non-coding RNA molecules that regulate gene expression and determine protein levels in cells by post-transcriptional repression (by target destabilization, translational inhibition, or both), with each individual miRNA possibly having dozens of targets^26–28^. They target messenger RNAs (mRNAs), thereby reducing protein levels of multiple proteins and hence are considered to fine-tune complex gene regulatory networks in the brain^29–31^. Dysregulation of miRNA levels is believed to contribute to the pathogenesis of neurological diseases, potentially delivering new diagnostic biomarkers and new therapeutic targets^32–36^. There is strong evidence for an association of miRNA dysregulation with epilepsy, and that protein levels of important signaling proteins in injured brain tissues are affected by differentially expressed miRNAs^1^. In addition, miRNA expression changes revealed differences in each stage of epilepsy development, particularly after the epileptogenic insult, on the day of the first spontaneous seizure, and during the chronic epilepsy phase (the period most clinically relevant when using miRNA-based therapeutics)^37^.

Moreover, miRNAs possess characteristics that make their targeting feasible for drug development, notably the in vivo delivery of these drugs through approved administration regimens^38,39^. Several pharmaceutical companies use miRNAs as drug targets, and such strategies have entered human trials^40^. Strategies to manipulate miRNAs in vivo include the use of genetic and oligonucleotide-based approaches^1,41,42^. Antagomirs are oligonucleotide miRNA inhibitors that have been shown to control seizures in vivo deployment^43,44^. Administration of antagomirs inhibiting miR-34a, miR-132, miR-134, and miR-184 resulted in the reduction of neuronal death caused by SE^21,45–48^. In this study, findings from a previous systematic in vivo screening approach of commonly altered microRNAs in TLE, as well as their regulation resulting in the reduction in seizure severity and burden during SE and significant anti-epileptic activity after SE were explored^37^.

miR-21a-5p was found to be up-regulated by at least 15% in the rodent models when targeted, suppressing seizures^37,49^. miR-142a-5p taken from serum samples of TLE patients was also revealed as a diagnostic biomarker, having an upregulated expression pattern^50^. Its inhibition results in the overexpression of its target genes, which then attenuates apoptosis or cell death, reduces neuronal damage in the hippocampus, promotes mitochondrial autophagy, and weakens SE in epileptic rats^51,52^. Although miR-10a-5p has limited functional data linked to epilepsy, it was also shown to be upregulated in different mouse models during the chronic epilepsy phase, and together, these upregulated epilepsy-associated miRNAs are fully conserved in humans and have been designed antagomirs^37^. Meanwhile, the antagomirs anti-miR-21a-5p, anti-miR-142a-5p, and anti-miR-10a-5p were observed to have anti-seizure effects, and transforming growth factor-*β* (TGF-*β*) signaling was identified as a shared mechanism between the targeted seizure-modifying miRNAs, extending the experimental setup by injecting the TGF-*β* pathway inhibitor galunisertib along with the antagomirs to induce an increase in seizure severity and to prove the role of TGF-*β* signaling in antagomir-based therapeutics^37^. TGF-*β* is a pleiotropic growth factor that is upregulated in many epileptogenic conditions^53–56^. It signals via binding to its receptors, TGF-*β*RI and TGF-*β*RII, activating the phosphorylation of SMAD2 and SMAD3, which associate with SMAD4 before being translocated to the nucleus to regulate transcriptional responses^57,58^. The upregulation of TGF-*β* signaling mediates seizure control and promotes an *anti-seizure state*, leading to the conclusion that TGF-*β* signaling regulation by miRNAs represents an interesting therapeutic target^37^.

Mathematical modeling has been used to describe the characteristic features of epileptogenesis, cellular responses related to epilepsy, internal time processing, and to examine different mechanisms that allow for the discovery of treatment strategies for epilepsy, particularly TLE^59,60^. Hybrid models for TGF-*β*/SMAD signaling pathways were used to investigate the diffusion/reaction in the extracellular microenvironment, protein/receptor surface interactions, crosstalk analysis with other signaling pathways, and intracellular processes represented as ordinary differential equations (ODEs) to interpret the changes in cellular response with varying TGF-*β* and SMAD concentrations^61,62^. Based on available research, no mathematical study exploring the underlying mechanisms of TGF-*β*/SMAD mediation of seizure control by miRNA regulation has been conducted. In this paper, a mathematical model has been developed for miRNA-regulated TGF-*β*/SMAD pathway analysis through inhibition by antagomirs in controlling seizure occurrences and characterizing both *seizure state* and *anti-seizure state*. The intracellular network consists of ODEs representing each component as variables, i.e., concentrations of (1) anti-miR-21a-5p, (2) anti-miR-142a-5p, (3) anti-miR-10a-5p, (4) miR-21a-5p, (5) miR-142a-5p, (6) miR-10a-5p, (7) TGF-*β*, and (8) SMAD. This study aims to investigate the (i) structure of miRNA/TGF-*β*/SMAD regulatory model, (ii) how variations in antagomir injections affect the concentration of intracellular components and the occurrence/non-occurrence of seizures, and (iii) verify treatment strategies in TLE based on the antagomir administration approach for potential therapeutic targeting of miRNAs. Non-dimensionalisation of model parameters, characterization of both *seizure state* and *anti-seizure state*, equilibrium analysis to investigate the concentration behavior of the dynamical system components, and sensitivity analysis on the main model variables were performed to give a better understanding of the intracellular dynamics and structure, as well as the cellular response to stimuli by antagomir injection.

## Results

### Model development

**Fig. 1:**
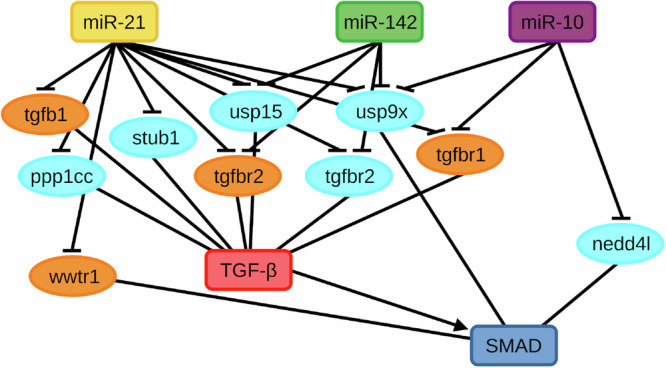
Wiring diagram of the dysregulated genes and miRNAs associated with TGF-*β* signaling. The schematic diagram for the statistically significant downregulated genes described in Fig. 9a targeted by the three seizure-modifying miRNAs: miR-21a-5p (yellow rounded rectangle), miR-142a-5p (green rounded rectangle), and miR-10a-5p (purple rounded rectangle) described in Fig. 9b and are involved in the enriched TGF-*β*/SMAD signaling pathways: signaling by TGF-*β* receptor complex (red rounded rectangle) and transcriptional activity of SMAD2/3:4 heterotrimer (blue rounded rectangle) as a simplified functional network. Significantly dysregulated mRNAs (orange ellipses) and proteins (cyan ellipses) are targeted by at least one miRNA and involved in either TGF-*β* or SMAD.

Simulation results of the mathematical model for seizure regulation in TLE were presented which is composed of key molecules in the intracellular network as shown in Fig. 1. These are the epilepsy-associated miRNAs: (1) miR-21a-5p (yellow rounded rectangle), (2) miR-142a-5p (green rounded rectangle), and (3) miR-10a-5p (purple rounded rectangle), and the components of the TGF-*β*/SMAD signaling pathways enriched in their targets: (4) TGF-*β* receptor complex (red rounded rectangle), and the downstream (5) transcriptional activity of SMAD2/3:4 heterotrimer (blue rounded rectangle). Taking only the pertinent network components from Venø et al., Fig. 1 also shows the wiring diagram of the intracellular molecules as a basis for mathematically modeling the seizure-modifying mechanism^37^. It illustrates the interactions between the seizure-modifying miRNAs and their targeted mRNAs—TGFBR1, TGFB1, TGFBR2, and WWTR1 (orange ellipses) and proteins—PPP1CC, USP15, STUB1, TGFBR2, USP9X, and NEDD4L (cyan ellipses), all associated with TGF-*β* signaling, where arrows and hammerheads in the diagram represent activation and inhibition, respectively.

### Characterization of seizure phenotypes

In this section, temporal dynamics of the intracellular activities of miR-21a-5p (*R*_1_), miR-142a-5p (*R*_2_), miR-10a-5p (*R*_3_), TGF-*β*(*T*), and SMAD (*S*) under antagomir injections anti-miR-21a-5p (*A*_1_), anti-miR-142a-5p (*A*_2_), and anti-miR-10a-5p (*A*_3_) are explored. Following the same experimental setup from Venø et al., the seizure treatment starts at *t* = −24 h where the antagomirs are pre-injected, then at *t* = 0 h where Kainic Acid is injected to induce seizures, and at *t* = 24 h when the brain is analyzed for gene expressions and protein concentrations^37^. The interval *t* = [−24 h, 0 h] is denoted as the *control phase*, while *t* = [0 h, 24 h] is called the *chronic phase* under which the upregulation or downregulation of the intracellular signaling dynamics will be evaluated. Following this, the two treatment strategies are then denoted as (1) antagomir injection and (2) scrambled control to examine their regulatory effects on seizure intracellular signaling dynamics and phenotype characterization in TLE.

**Fig. 2:**
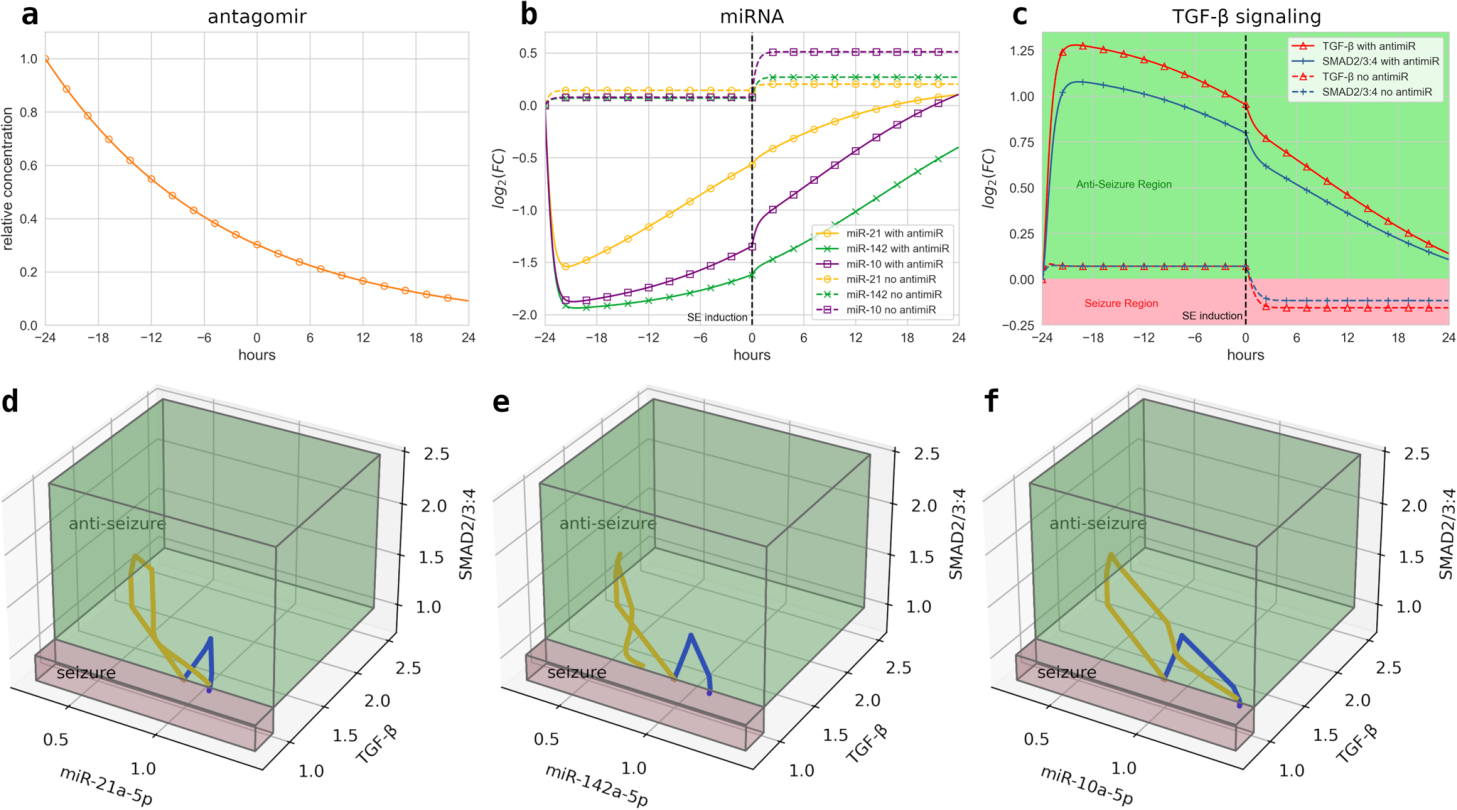
2-D temporal plots and 3-D trajectories of model components. Time series plots for **a** antagomirs, **b** miRNAs, and the **c** TGF-*β*/SMAD signaling pathways evaluated from antagomir pre-injection at *t* = −24 h up to the Western blot analysis at *t* = 24 h under both antagomir (solid lines) and scrambled control (dashed lines) pre-injections. Seizure (red area) and anti-seizure (green area) regions are shaded accordingly, with concentration trajectories measured in ${\log }_{2}$(fold change (FC) over control). Three-dimensional concentration trajectories of **d** miR-21a-5p, **e** miR-142a-5p, and **f** miR-10a-5p versus TGF-*β* and SMAD under both antagomir pre-injection (orange curves) and scrambled control (blue curves) strategies. Concentrations are measured in FC over control.

Figure 2a shows the initial bolus antagomir injection dosage for *A*_1_–*A*_3_ (orange solid lines) was defined with a relative concentration of 1.0, which decays over the treatment duration. The trajectories in Fig. 2b show the curves for *R*_1_ (yellow lines), *R*_2_ (green lines), and *R*_3_ (purple lines) under both the antagomir (solid lines) and control (dashed lines) treatments over time *t*. Without antagomir pre-injections, small growths in miRNA levels can be observed before stabilizing to steady states within the *control phase*, which again increase before stabilizing upon SE induction in the *chronic phase*. However, in response to antagomir influence, miRNA levels drastically decrease before steadily growing in the *control phase*, and continuing their growth after SE induction until the end of the *chronic phase*. In Fig. 2c, the concentration evolutions of *T* (red lines) and *S* (blue lines) in response to both the antagomir (solid lines) and control (dashed lines) treatments over time *t* are shown. A small increase in levels for *T* and *S* could be observed before stabilizing to steady states when under no antagomir pre-injections within the *control phase*. However, the concentrations drop below threshold levels before stabilizing after SE induction in the *chronic phase*. Meanwhile, in the *control phase*, *T* and *S* levels rapidly increase under antagomir administration as a consequence of the drastic drop in miRNA levels. The levels then steadily decrease through SE induction and towards the end of the *chronic phase*. The phenotypic threshold can also be seen to divide the red and green regions for the *T* and *S* curves to characterize the system into seizure and *anti-seizure state*, respectively. Furthermore, Fig. 2d–f shows the three-dimensional curves for the concentrations of all three epilepsy-associated miRNAs with respect to *T* and *S* levels. It can be observed that trajectories are located in both seizure (red area) and anti-seizure (green area) regions when no antagomirs are pre-injected (blue curves). Otherwise, the trajectories are only confined in the anti-seizure region when antagomirs are administered (orange curves) 24 h prior to SE induction.

**Fig. 3:**
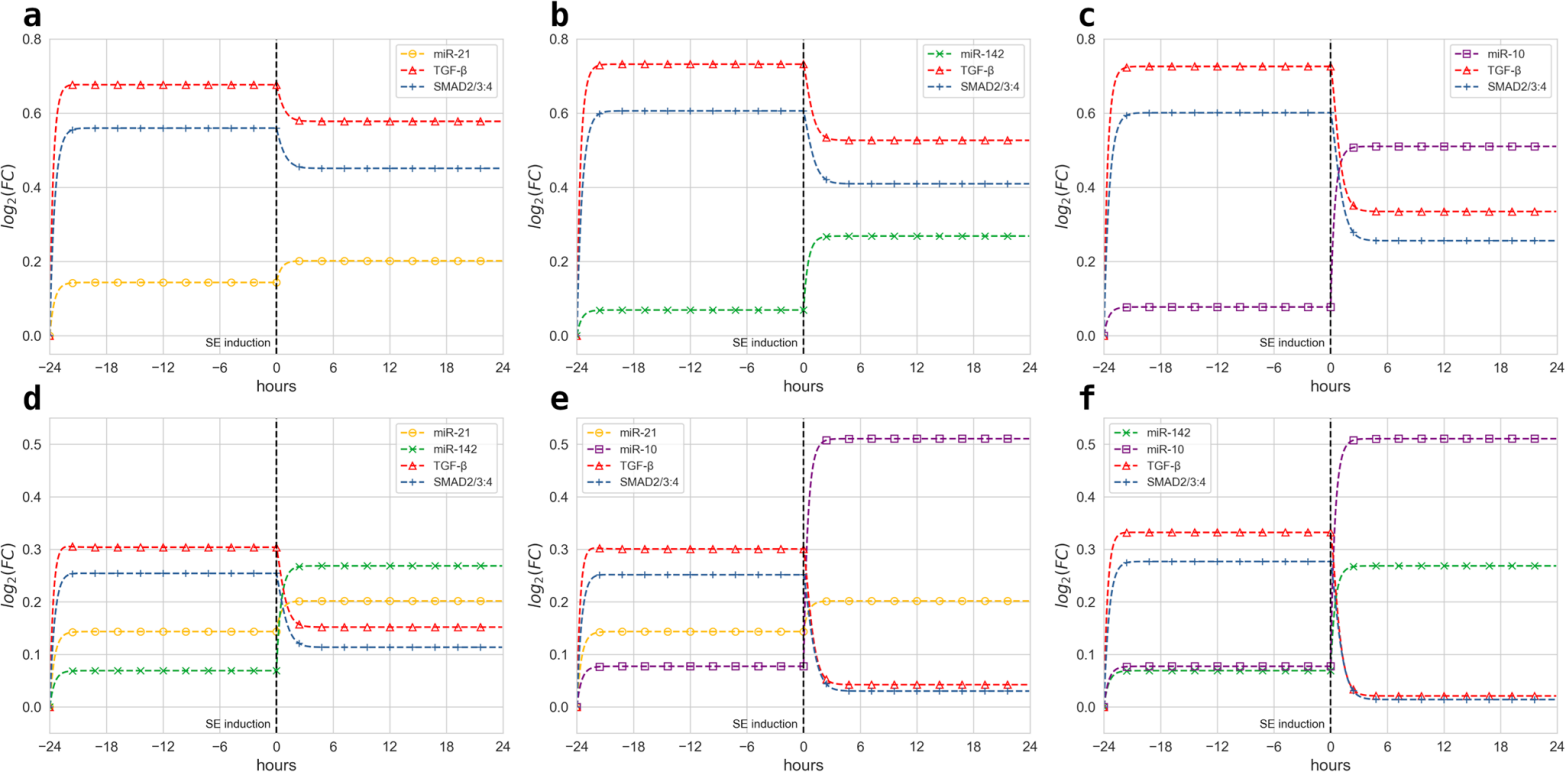
Temporal plots of TGF-*β* signaling under different miRNA combinations. Temporal evaluations of concentration trajectories of the intracellular components without antagomir administration for the downstream TGF-*β*/SMAD signaling pathways regulated individually by **a** miR-21a-5p, **b** miR-142a-5p, or **c** miR-10a-5p. Similar trajectory plots for the regulation of the TGF-*β*/SMAD signaling pathways under the miRNA combinations: **d** miR-21a-5p and miR-142a-5p, **e** miR-21a-5p and miR-10a-5p, and **f** miR-142a-5p and miR-10a-5p. All plots are evaluated from antagomir pre-injection at *t* = −24 h up to the Western blot analysis at *t* = 24 h. Concentration plots are measured in ${\log }_{2}$(FC over control).

The model dynamics and outcome under different model structures are then examined, particularly investigating each individual and combined effects of the epilepsy-associated miRNAs on the downstream TGF-*β*/SMAD signaling pathways, as illustrated in Fig. 3. Each miRNA targets significantly dysregulated mRNAs and proteins that are associated with either the TGF-*β* receptor complex or the SMAD2/3:4 heterotrimer as shown in Fig. 1. Hence, Fig. 3a–c illustrates the three constructed dynamical systems to disjointedly model the inhibitory effects of *R*_1_ (yellow dashed lines), *R*_2_ (green dashed lines), and *R*_3_ (purple dashed lines) concentrations on *T* (red dashed lines) and *S* (blue dashed lines) levels without antagomir influence. The regulation by individual miRNAs of their downstream TGF-*β* and SMAD led to an observed increase in miRNA levels during the *control phase* due to normal growth from their signaling source. Thereafter, upon SE induction, each miRNA was seen to be further up-regulated as a response to seizure activity in the system during the *chronic phase*. As a result, the downstream *T* and *S* levels are downregulated due to inhibition by each miRNA. In Fig. 3d–f, three dynamical systems for each pairwise combination of *R*_1_ (yellow dashed lines), *R*_2_ (green dashed lines), and *R*_3_ (purple dashed lines) were constructed to explore the different downstream *T* (red dashed lines) and *S* (blue dashed lines) regulatory profiles under scrambled controls. The same trajectories for all miRNAs in each setup were expected; however, it can be noticed that *T* and *S* levels are significantly lower than when under the inhibition of a single miRNA. This is due to the combined inhibitory effect of any two miRNAs during the *control phase*. It then follows in a similar fashion that the inhibition by two miRNAs results in more down-regulation of *T* and *S* levels, which is very distinguishable compared to down-regulated *T* and *S* levels inhibited by a single miRNA.

**Fig. 4:**
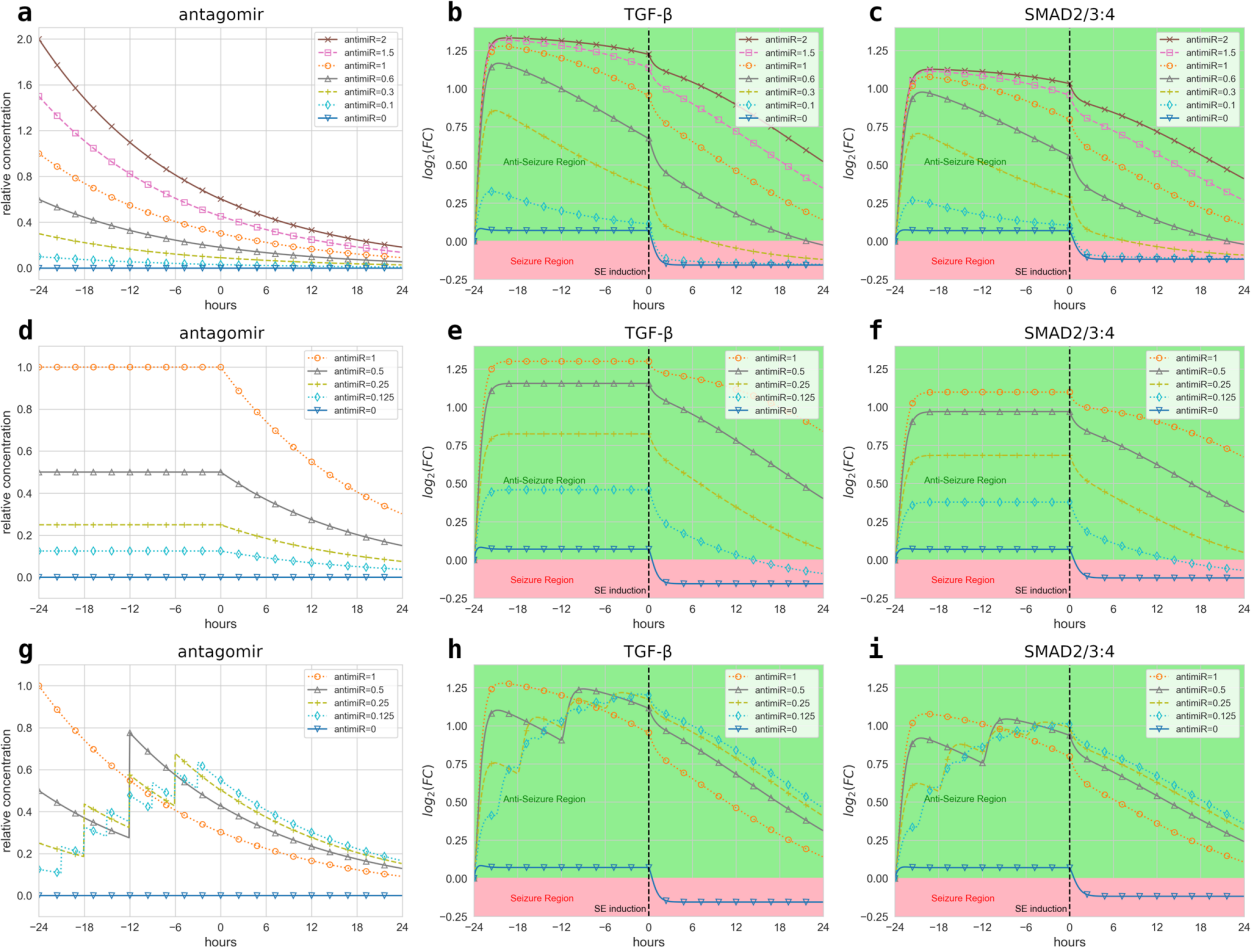
Temporal plots of TGF-*β* signaling under different antagomir administration strategies. Temporal evaluations of the model component concentrations under the bolus antagomir pre-injection strategy under **a** varying initial antagomir dosages and their resulting **b** TGF-*β* and **c** SMAD curves. Concentration plots of the model components under the continuous antagomir infusion strategy showing the **d** continuous antagomir infusions at varying levels and their resulting **e** TGF-*β* and **f** SMAD curves. Time series plots for the model trajectories under the multiple antagomir dosage strategy showing the **g** antagomir dosages distributed into varying injection frequencies within the initial 24 h and their resulting **h** TGF-*β* and **i** SMAD curves. All plots are evaluated from antagomir administration starting time at *t* = −24 h up to the Western blot analysis at *t* = 24 h. Seizure (red area) and anti-seizure (green area) regions are shaded accordingly, with concentration plots measured in ${\log }_{2}$(FC over control).

At this point, several proposed antagomir administration strategies were then explored to investigate different phenotypic switching scenarios with a goal of promoting the system in an *anti-seizure state* as shown in Fig. 4. The first of the proposed administration strategies is denoted as the *bolus pre-injection strategy* which is defined by administering a single antagomir dosage 24 h prior to SE induction at the start of the *control phase* for all antagomirs inhibiting each target miRNA, in which its concentration decays over the treatment duration. Simulations were performed under varying initial antagomir dosages shown in Fig. 4a and their resulting TGF-*β* and SMAD profiles depicted in Fig. 4b, c, respectively. Increasing antagomir pre-injection dosages were generally observed, which resulted in higher antagomir relative concentrations over time *t* and led to increased *T* and *S* concentrations for the entire treatment duration. Plots also show that antagomir dosages *A*_1_ = *A*_2_ = *A*_3_ = 0.6 or lower are unable to sustain the system in a healthy state and switch to the *seizure state* (red area), while antagomir dosages *A*_1_ = *A*_2_ = *A*_3_ = 1.0 can keep it in an *anti-seizure state* (green area) with respect to *T* and *S* concentrations. These results suggest that antagomir pre-injection dosages can be chosen to produce the desired phenotype that the system could promote. Next, another administration strategy was explored called the *continuous infusion strategy*, defined by administering an antagomir infusion for the duration of the *control phase* while maintaining its concentration levels for each antagomir and its target miRNAs. Simulations were also performed under various sustained antagomir concentration levels for 24 h prior to SE induction, as shown in Fig. 4d, and their corresponding TGF-*β* and SMAD profiles are illustrated in Fig. 4e, f, respectively. Looking at the plots, decreasing the maintained antagomir concentration levels in the *control phase* was observed to reduce the antagomir levels in the *chronic phase*, which also reduced the yielded *T* and *S* concentrations. Interestingly, the simulations also revealed that relative antagomir infusion levels of *A*_1_ = *A*_2_ = *A*_3_ = 0.25 or higher were able to keep the model trajectories confined in the *anti-seizure state* and thus, promote the system in a healthy state. Conversely, relative antagomir infusion levels of *A*_1_ = *A*_2_ = *A*_3_ = 0.125 or lower were unable to sustain the system in a healthy state and prompted it to switch to the *seizure state*. Lastly, the final proposed antagomir administration strategy is called the *multiple dosage strategy*, which was created by dividing the standard single antagomir injection dosage into equal parts on various administration frequencies at equal intervals during the *control phase* for all three antagomirs and the miRNAs they inhibit. The antagomir dosage of *A*_1_ = *A*_2_ = *A*_3_ = 1.0 was divided across various injection frequencies for the first 24 h before SE induction, where antagomir injections similarly decay and succeeding dosages would add to the concentration level at the next injection time as shown in Fig. 4g. Simulations then revealed that the produced TGF-*β* and SMAD profiles in Fig. 4h, i, respectively, display sustained concentration levels during the *chronic phase* and keep the system in an *anti-seizure state*. However, the plots interestingly exhibited higher sustained *T* and *S* levels in the *chronic phase* for simulations with higher administration frequencies and thus, multiple antagomir injections at lower dosages than that of the standard single antagomir pre-injection strategy. This suggests that increasing the frequency of antagomir injections without necessarily running a risk of an overdose can further upregulate *T* and *S* concentrations, thus also keeping the system in an *anti-seizure state*.

**Fig. 5:**
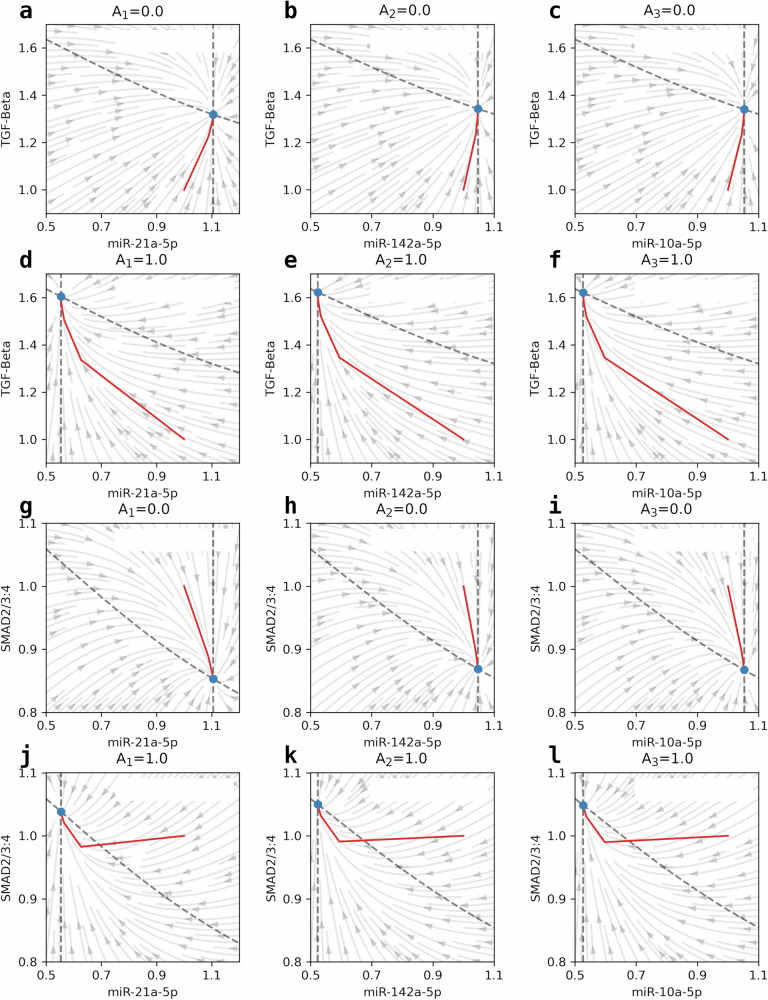
Behavioral analysis of the seizure regulatory model as a dynamical system. Nullclines and phase plane diagrams for TGF-*β* with respect to **a** miR-21a-5p, **b** miR-142a-5p, and **c** miR-10a-5p under scrambled controls (*A*_1_ = *A*_2_ = *A*_3_ = 0.0) describing the solution flow and the steady state behaviors of the model, as well as for **d** miR-21a-5p, **e** miR-142a-5p, and **f** miR-10a-5p under antagomir injections (*A*_1_ = *A*_2_ = *A*_3_ = 1.0) Also shown are **b** nullclines and phase plane diagrams for SMAD with respect to **g** miR-21a-5p, **h** miR-142a-5p, and **i** miR-10a-5p when *A*_1_ = *A*_2_ = *A*_3_ = 0.0 and for **j** miR-21a-5p, **k** miR-142a-5p, and **l** miR-10a-5p when *A*_1_ = *A*_2_ = *A*_3_ = 1.0. Stable nodes (blue dots), nullclines (gray lines), phase planes (gray arrow field), and concentration trajectories (red lines) can be seen in each plot.

The equilibrium points, nullclines, and phase planes in Fig. 5a–c are used to describe the different intracellular states of *T* versus *R*_1_–*R*_3_ levels under scrambled controls (antagomir relative dosages at *A*_1_ = *A*_2_ = *A*_3_ = 0.0 promoting a *seizure state*), and under antagomir pre-injections 24 h before SE induction (antagomir relative dosages at *A*_1_ = *A*_2_ = *A*_3_ = 1.0 promoting a *anti-seizure state*) shown in Fig. 5d–f. Moreover, Fig. 5g–i illustrates the behavior of *S* trajectories in response to scrambled controls, while Fig. 5j–l describes the dynamics under the antagomir treatment. Stable nodes (blue dots) in Fig. 5a, l are equilibrium points describing the local nature and stability of the equilibria, which, in this case, are the convergent solutions where the model trajectories (red curves) flow into as steady states in the dynamical system. These represent the model solution and validate the stability of the intracellular signaling dynamics, which is important in regulating the model and characterizing both *seizure state* and *anti-seizure state*. Additional behaviors of the system are provided by the phase diagrams drawn as arrow fields, which act as the direction and magnitude of the solution flow. Also shown in the plots are the nullclines, which are the set of points in the phase diagram where the rate of change is zero, or in other words, the space where possible steady state solutions of the model concentrations could be found. These results further establish the proposed characterization of both *seizure state* and *anti-seizure state*, which are fundamental in developing treatment strategies for seizures in TLE.

### Sensitivity analysis of the intracellular dynamics

The mathematical model described in (13)–(20) has a number of parameters with no available experimental data used to estimate their values, but they may still significantly affect the behavior of the model dynamics. This study also aims to focus on how different inhibition processes influence the resulting molecular concentrations. Sensitivity analysis was then performed for key parameters, particularly the inhibition strength parameters (*α*, *β*, *γ*, *δ*, *ϵ*, *ζ*, *η*, *θ*, and *κ*) at the brain analysis time point (*t* = 24 h). This determines which inhibition processes have greater impacts on the model outcome, thus proposing new regulatory techniques for epilepsy-associated miRNAs and the downstream TGF-*β* signaling pathways. Future therapeutic targets for seizure control in TLE could be identified besides the proposed strategy in this work to maintain the system in an *anti-seizure state*, thereby reducing seizure occurrence, which essentially is the aim in this study.

Extended Fourier amplitude sensitivity test (eFAST) based on the original FAST is a variance decomposition method where perturbations are applied to input parameters which causes model output variations. After quantifying the variations similar to statistical variances, the algorithm partitions the output variance into fractions explained by each input parameter by varying them at different frequencies. The strengths of these parameter frequencies in the model output are then measured using Fourier analysis, which basically serves as the sensitivity measure of a model to the parameter. eFAST then calculates two sensitivity indices: (1) the fraction of the variance explained by the perturbation of a single input parameter called the first-order sensitivity index *S*_1_, and (2) the fraction which includes all higher order and nonlinear interactions between the input parameter and the other remaining parameters called the total-order sensitivity index *S*_*T*_, where *S*_1_, *S*_*T*_ ∈ [0, 1]^63^. A sample size of *N* = 1000 runs was chosen in performing this sensitivity test, which was also used in other sensitivity tests on some mathematical models^64,65^.

**Fig. 6:**
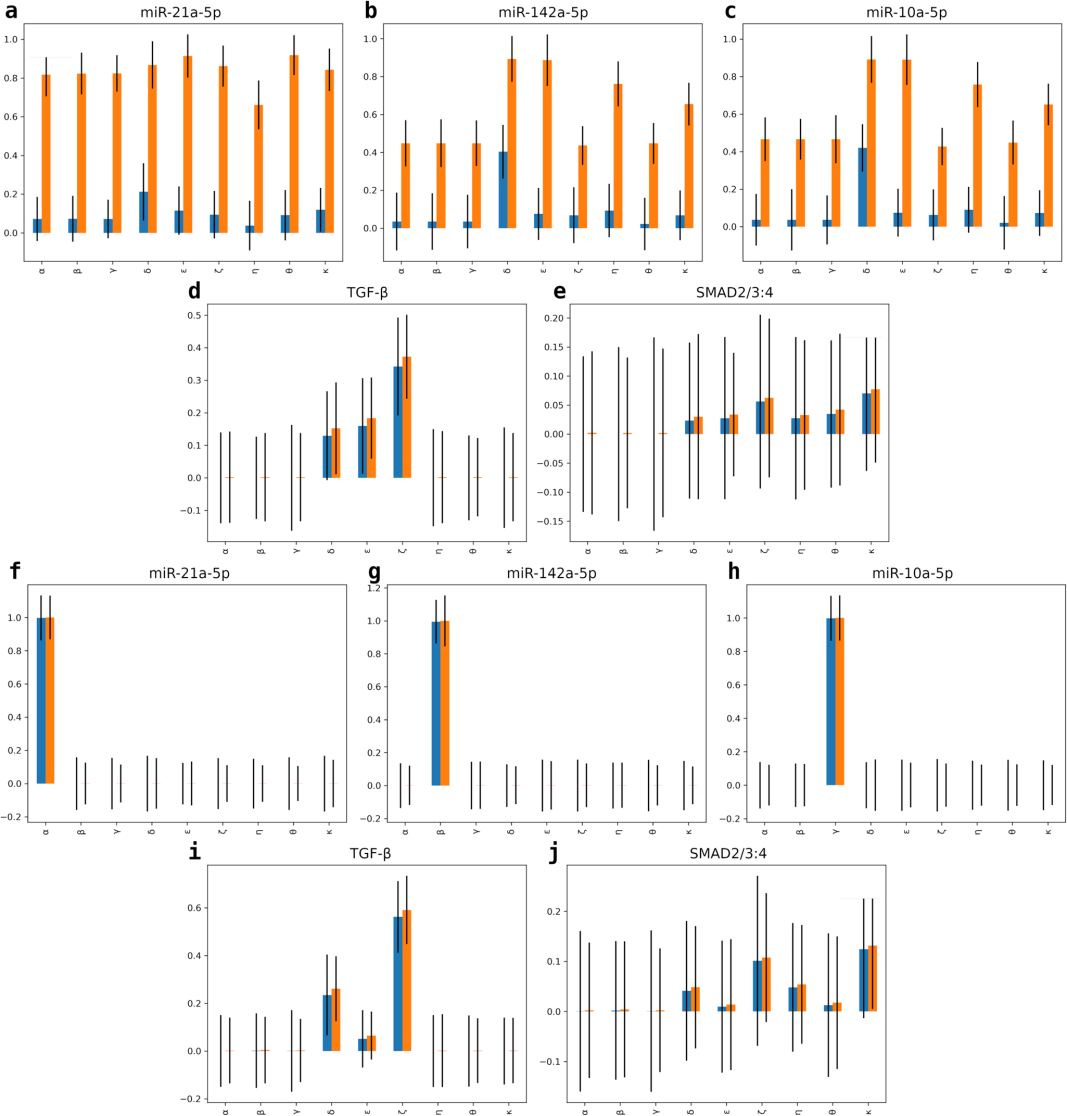
Sensitivity analysis of the seizure regulatory model to test robustness. Extended Fourier amplitude sensitivity test (eFAST) first-order *S*_1_ (blue bars) and total-order *S*_*T*_ (orange bars) sensitivity indices of the model components **a** miR-21a-5p, **b** miR-142a-5p, **c** miR-10a-5p, **d** TGF-*β*, and **e** SMAD across inhibition strength parameters *α*, *β*, *γ*, *δ*, *ϵ*, *ζ*, *η*, *θ*, and *κ* under the control (scrambled controls) treatment strategy with error bars overlaid to represent a 95% confidence interval. Also illustrated are plots of eFAST *S*_1_ (blue bars) and *S*_*T*_ (orange bars) sensitivity indices for **f** miR-21a-5p, **g** miR-142a-5p, **h** miR-10a-5p, **i** TGF-*β*, and **j** SMAD across inhibition strength parameters under the antagomir (antagomir pre-injections) treatment strategy.

Observations from Fig. 6a–c revealed higher *S*_*T*_ (orange bars) values for *R*_1_–*R*_3_ than their *S*_1_ (blue bars) values under scrambled controls (without antagomir pre-injections), implying that miRNA inhibition is more sensitive to the effects of other parameters in the model than the particular inhibition strength parameter. This is attributed to the stochastic nature of eFAST as the model is under no antagonist pre-injections. Meanwhile, Fig. 6d, e for the signaling pathways *T* and *S* levels, respectively, have almost equal *S*_1_ and *S*_*T*_ indices only for related parameters describing their involved inhibition processes: *δ*, *ϵ*, and *ζ* for *T* representing the inhibition strengths on TGF-*β* by the seizure-modifying miRNAs and, in addition to these, *η*, *θ*, and *κ* for *S* as the inhibition strengths on SMAD. These values are expected since antagomir injections that would have inhibited the miRNAs affecting these two downstream signaling pathways are not present, limiting the resulting output variance for *T* and *S* to be only caused by the perturbations of parameters related to their inhibiting upstream pathways. On the other hand, Fig. 6f–h showed both *S*_1_ and *S*_*T*_ indices of the seizure-modifying miRNAs to be similar and sensitive for the inhibition strength parameter related to them when under antagomir pre-injections. This implies that miRNA inhibition is only sensitive to the effects of how strongly the antagomir inhibits their targeted miRNA, which is also the expected behavior. In addition, *T* and *S* concentrations under the antagomir treatment were found to be sensitive to the variations of the same inhibition strength parameters when the system is under the control treatment, as displayed in Fig. 6i, j, respectively. Interestingly, *ζ* has the highest sensitivity index value, followed by *δ*, with *ϵ* close behind for *T* and additionally, *κ* is the highest with *η* and *θ* further down for *S* under scrambled control injections. This translates to *R*_3_ apparently having more effect on the output variation of *T* and *S* levels, which is also only around half as sensitive to the inhibition by both *R*_1_ and *R*_2_ when no antagomirs are administered. However, under antagomir treatments, *T* and *S* become the least sensitive to inhibition by *R*_2_, with inhibition strengths by *R*_1_ and *R*_3_ still having around the same sensitivity index values as without antagomirs pre-injected. These results from the performed sensitivity analysis may suggest the choice of antagomir to be used to block inhibitive pathways of TGF-*β* and SMAD, thereby upregulating their expression levels to keep the system in the *anti-seizure state*. This will provide insights into developing novel seizure severity and burden reduction strategies in TLE.

## Discussion

This work builds on a previous experimental study that applied miRNAs and their targeted mRNAs and proteins involved in TGF-*β* signaling for seizure suppression in TLE. A mathematical model of the associated intracellular signaling networks was developed to investigate the mechanisms of miRNA inhibition via antagomirs, providing a systems-based framework for designing targeted seizure treatment strategies based on the work by Venø et al.^37^. The model consists of antagomirs that are injected into the brain (intracerebroventricular administration) to inhibit their target miRNAs and genes associated with the TGF-*β*/SMAD signaling pathways, where ODEs were used to represent the reaction rate equations for each model component, with the goal of controlling the concentrations of seizure-modifying miRNAs and its downstream signaling pathways through antagomir injections.

Based on the experimental setup in Venø et al., the treatment strategy where antagomirs were pre-injected 24 h before SE induction and Western blot analysis was performed 24 h afterwards was explored to investigate the characterization of the seizure and anti-seizure phenotypes in more detail^37^. This strategy is then compared to the control treatment strategy with the system under scrambled controls instead. Under both strategies, the model was initialized in an *anti-seizure state* and found the treatment strategy to keep the system in the same *anti-seizure state*, whereas the control strategy enabled the system to switch into the *seizure state*. Examination of the model under high antagomir dosages revealed down-regulated miRNA concentrations and improved mediation by TGF-*β* signaling, promoting the system in an *anti-seizure state*, whereas low antagomir dosages interestingly resulted in up-regulated miRNA concentrations and repressed TGF-*β* signaling, promoting the system in a *seizure state*. Numerical simulations performed on the mathematical model to characterize the seizure and anti-seizure phenotypes supported the comparative analysis between the two strategies. Examining the individual and pairwise regulation of each miRNA independent from any antagomir influence also revealed differing concentration profiles for the downstream TGF-*β*/SMAD signaling pathways, thus demonstrating the impact of the model structure on the mechanisms affecting the phenotype switching. Three antagomir administration strategies were then considered, namely the bolus pre-injection, continuous infusion, and multiple dosage strategies, suggesting various approaches in keeping the system in an *anti-seizure state*, therefore suppressing seizures. These results altogether validate the phenotypic switching in response to the presence or absence of antagomirs in the system, as well as the anti-seizure effects of antagomirs, thereby providing key insights into potential therapeutic targets for seizure suppression, particularly in TLE.

This work is a first step toward combining experimental and modeling approaches for new seizure suppression strategies in TLE through a detailed analysis of intracellular signaling dynamics. Future recommendations include exploring other modeling approaches, such as exploring artificial intelligence (AI) models, designing strategies to address other pathological responses in TLE, and modeling using experimental data on other microenvironmental components, such as transfer RNA-derived small RNAs (tsRNAs), circular RNAs (circRNAs), and other related signaling pathways. Also worth noting in the future are post-treatment strategies wherein antagomirs are injected only after SE induction^37^.

## Methods

### Signaling pathway regulation

The concentrations for miR-21a-5p, miR-142a-5p, miR-10a-5p, TGF-*β*, and SMAD are then denoted as ${\overline{R}}_{1}$, ${\overline{R}}_{2}$, ${\overline{R}}_{3}$, $\overline{T}$, and $\overline{S}$ at time $\overline{t}$, respectively. Therefore, the mass balance of the concentrations for ${\overline{R}}_{1}$, ${\overline{M}}_{2}$, ${\overline{M}}_{3}$, $\overline{T}$, $\overline{S}$ gives us

1 $$\frac{{\rm{d}}{\bar{R}}_{1}}{{\rm{d}}\bar{t}}=\mathop{\underbrace{{f}_{\mathrm{miR}-21}}}\limits_{\mathrm{source}}+\mathop{\underbrace{\frac{{\tau }_{1}{\phi }_{1}^{2}}{{\phi }_{1}^{2}+{\upsilon }_{1}{G}_{1}({\bar{A}}_{1})}}}\limits_{{\bar{A}}_{1} \dashv {\bar{R}}_{1}}-\mathop{\underbrace{{\mu }_{\mathrm{miR}-21}{\bar{R}}_{1}}}\limits_{\mathrm{decay}}$$

2 $$\frac{{\rm{d}}{\bar{R}}_{2}}{{\rm{d}}\bar{t}}=\mathop{\underbrace{{f}_{\mathrm{miR}-142}}}\limits_{\mathrm{source}}+\mathop{\underbrace{\frac{{\tau }_{2}{\phi }_{2}^{2}}{{\phi }_{2}^{2}+{\upsilon }_{2}{G}_{2}({\bar{A}}_{2})}}}\limits_{{\bar{A}}_{2} \dashv {\bar{R}}_{2}}-\mathop{\underbrace{{\mu }_{\mathrm{miR}-142}{\bar{R}}_{2}}}\limits_{\mathrm{decay}},$$

3 $$\frac{{{\rm{d}}{{\bar R}_3}}}{{{\rm{d}}\bar t}} = \underbrace {{f_{{\rm{miR}} - 10}}}_{{\rm{source}}} + \underbrace {\frac{{{\tau _3}\phi _3^2}}{{\phi _3^2 + {\upsilon_3}{G_3}\left( {{{\bar A}_3}} \right)}}}_{{A_3} \dashv {{\bar R}_3}} - \underbrace {{\mu _{{\rm{miR}} - 10}}{{\bar R}_3}}_{{\rm{decay}}},$$

4 $$\frac{{\rm{d}}\overline{{\rm{T}}}}{{\rm{d}}\bar{t}}=\mathop{\underbrace{{f}_{\mathrm{TGF}-\beta }}}\limits_{\mathrm{source}}+\mathop{\underbrace{\frac{{\tau }_{4}{\phi }_{4}^{2}}{{\phi }_{4}^{2}+{\upsilon }_{4}{G}_{4}({\bar{R}}_{1},{\bar{R}}_{2},{\bar{R}}_{3})}}}\limits_{{\bar{R}}_{1} \dashv \bar{T},{\bar{R}}_{2} \dashv \bar{T},{\bar{R}}_{3} \dashv \bar{T}}-\mathop{\underbrace{{\mu }_{\mathrm{TGF}-\beta }\overline{T}}}\limits_{\mathrm{decay}},$$

5 $$\frac{d\overline{S}}{dt}=\mathop{\underbrace{{f}_{\mathrm{SMAD}}}}\limits_{\mathrm{source}}+\mathop{\underbrace{{\lambda }_{\mathrm{TGF}-\beta }\overline{T}}}\limits_{\overline{T}\to \overline{S}}+\mathop{\underbrace{\frac{{\tau }_{5}{\phi }_{5}^{2}}{{\phi }_{5}^{2}+{\upsilon }_{5}{G}_{5}({\overline{R}}_{1},{\overline{R}}_{2},{\overline{R}}_{3})}}}\limits_{{\overline{R}}_{1} \dashv \overline{S},{\overline{R}}_{2} \dashv \overline{S},{\overline{R}}_{3} \dashv \overline{S}}\,-\mathop{\underbrace{{\mu }_{\mathrm{SMAD}}\overline{S}}}\limits_{\mathrm{decay}},$$

where ${\bar{A}}_{1}$, ${\bar{A}}_{2}$, and ${\overline{A}}_{3}$ are concentrations of the antagomirs anti-miR-21a-5p, anti-miR-142a-5p, and anti-miR-10a-5p, at time $\overline{t}$, respectively, which function as miRNA inhibitors.

### microRNA inhibition

**Fig. 7:**
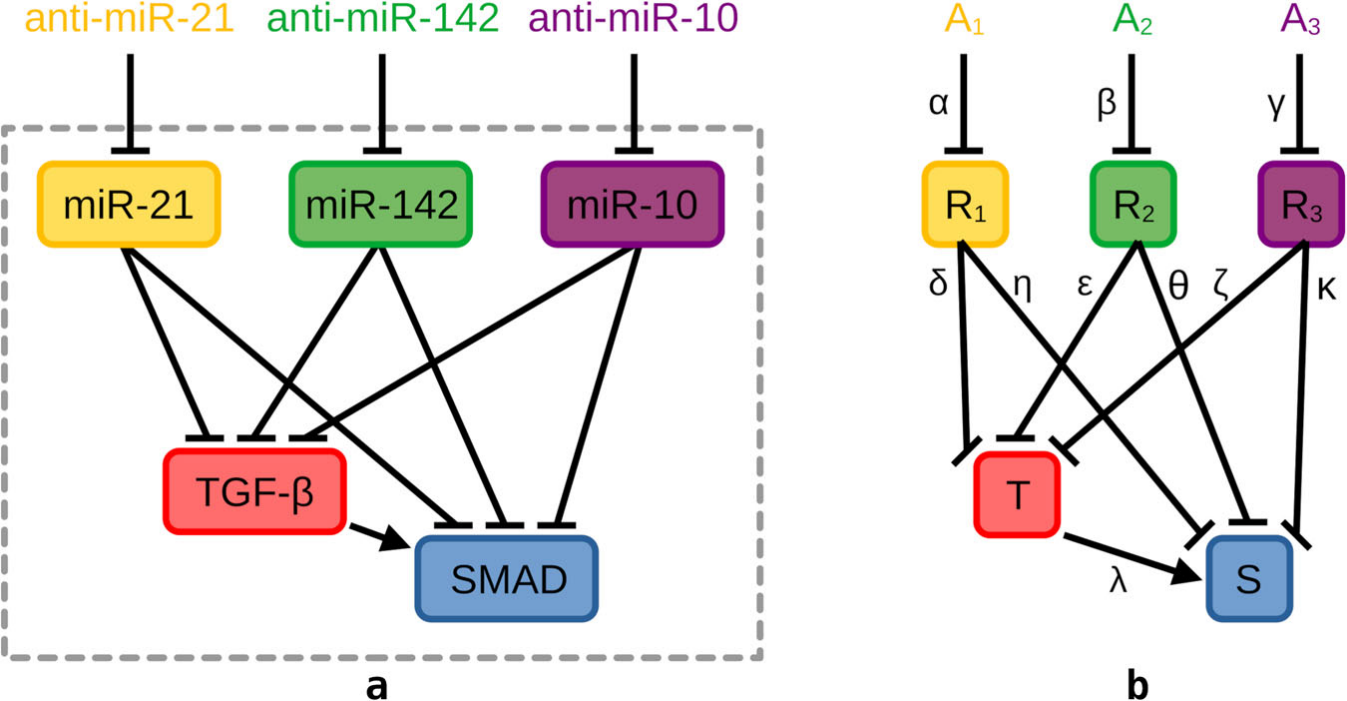
Model diagrams of the seizure regulation intracellular model. **a** Schematic diagram describing the model interactions where the antagomir injections anti-miR-21a-5p (yellow text), anti-miR-142a-5p (green text), and anti-miR-10a-5p (purple text) inhibit the seizure-modifying miRNAs miR-21a-5p (yellow rounded rectangle), miR-142a-5p (green rounded rectangle), and miR-10a-5p (purple rounded rectangle), which represses TGF-*β* signaling composed of the TGF-*β* receptor complex (red rounded rectangle) and its downstream pathway SMAD2/3:4 heterotrimer (blue rounded rectangle). Activations and inhibitions in the diagram are represented by arrows and hammerheads, respectively. **b** Derived diagram for the model equations with antagomirs, miRNAs, TGF-*β*, and SMAD denoted as variables, with the activation rates and inhibition strengths in Greek letters describing each interaction in the system.

In formulating the model, these antagomir injections target their corresponding miRNAs, resulting in their inhibition as shown in Fig. 7a. The effect of antagomirs on the inhibition of miRNAs and the downstream TGF-*β* signaling fundamentally proposes the seizure-modifying mechanism of the intracellular network model. This additionally gives us

6 $$\frac{{\mathrm{d}}{\overline{{A}}}_{1}}{{\mathrm{d}}\bar{t}}=-\mathop{\underbrace{{\mu }_{{\mathrm{anti}}-{\mathrm{miR}}-21}{\overline{A}}_{1}}}\limits_{{\mathrm{decay}}},$$

7 $$\frac{{\mathrm{d}}{\overline{A}}_{2}}{{\mathrm{d}}\bar{t}}=-\mathop{\underbrace{{\mu }_{{\mathrm{anti}}-{\mathrm{miR}}-142}{\overline{A}}_{2}}}\limits_{{\mathrm{decay}}},$$

8 $$\frac{{\mathrm{d}}{\overline{A}}_{3}}{{\mathrm{d}}\bar{t}}=-\mathop{\underbrace{{\mu }_{{\mathrm{anti}}-{\mathrm{miR}}-10}{\overline{A}}_{3}}}\limits_{{\mathrm{decay}}}.$$

**Fig. 8:**
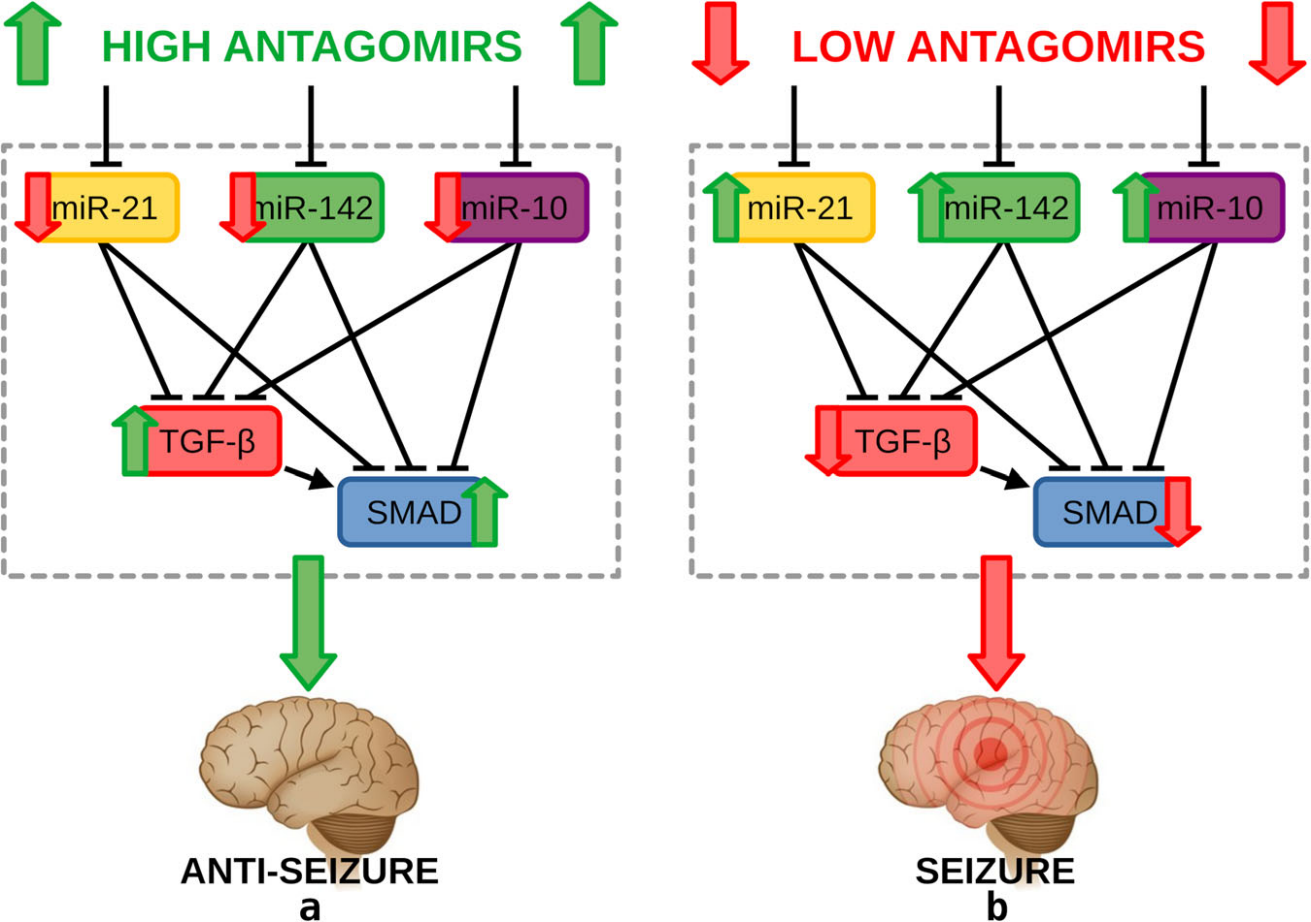
Network diagrams for the characterization of the two seizure phenotypes. **a** The proposed anti-seizure phenotypic regulation controlled by antagomir injections affecting the intracellular signaling dynamics. The intracellular response to high antagomir levels is downregulated miR-21a-5p (yellow rounded rectangle), miR-142a-5p (green rounded rectangle), and miR-10a-5p (purple rounded rectangle) concentrations and upregulated TGF-*β* (red rounded rectangle) and SMAD (blue rounded rectangle) levels, promoting an anti-seizure state. **b** The diagram representing the anti-seizure phenotype where low antagomir injection levels result in upregulated miRNA concentrations and down-regulated TGF-*β* signaling, allowing the system to switch into a seizure state.

Antagomir presence inhibits the concentrations of their targeted miRNAs, yielding downregulated levels for the three seizure-modifying miRNAs. These conditions of low miRNA concentrations in the intracellular space are insufficient to suppress TGF-*β* signaling, which results in upregulated TGF-*β* and SMAD concentrations. This TGF-*β* signaling upregulation then promotes the intracellular dynamics in an *anti-seizure state*, as well as a significant reduction in seizure severity, seizure burden, and spontaneous seizures^37^. This implies TGF-*β* signaling mediation by antagomir administration through targeted miRNAs as the underlying mechanism in promoting anti-seizure effects. On the other hand, antagomir absence leads to the overexpression of the three seizure-modifying miRNAs, resulting in upregulated miRNA concentrations, which then suppress the expression of TGF-*β* and SMAD. Correspondingly, this TGF-*β* signaling downregulation then promotes the system to enter the seizure phase. In this work, the model assumes a homogeneous distribution of intracellular miRNAs and signaling pathways across both *seizure state* and *anti-seizure state* of the brain. Figure 8a, b illustrates the two different intracellular states of the seizure regulatory model in response to high or low antagomir dosages, respectively. The indication of seizure phenotypes allows the model to regulate TGF-*β* (red rounded rectangle) and SMAD (blue rounded rectangle) levels as a predicted convergent mechanism of the seizure-modifying miRNAs miR-21a-5p (yellow rounded rectangle), miR-142a-5p (green rounded rectangle), and miR-10a-5p (purple rounded rectangle), thus revealing potential therapeutic targets for seizure suppression in TLE.

### Non-dimensionalisation and governing equations

The rate changes for antagomir injections ${\overline{A}}_{1}$, ${\overline{A}}_{2}$, and ${\overline{A}}_{3}$ are composed of the natural decay of their levels ${\mu }_{{\rm{a}}{\rm{n}}{\rm{t}}{\rm{i}}-{\rm{m}}{\rm{i}}{\rm{R}}-21}{\overline{A}}_{1}$, ${\mu }_{{\rm{a}}{\rm{n}}{\rm{t}}{\rm{i}}-{\rm{m}}{\rm{i}}{\rm{R}}-142}{\overline{A}}_{2}$, and ${\mu }_{{\rm{a}}{\rm{n}}{\rm{t}}{\rm{i}}-{\rm{m}}{\rm{i}}{\rm{R}}-10}{\overline{A}}_{3}$ as shown in (6)–(8). The rate changes for miRNA concentrations ${\overline{R}}_{1}$, ${\overline{R}}_{2}$, and ${\overline{R}}_{3}$ are also regulated by the signaling sources expressed as functions at fixed rates *f*_miR-21_, *f*_miR-142_, and *f*_miR-10_, autocatalytic activities with their inhibition by the antagomirs (${\overline{A}}_{1} \dashv {\bar{R}}_{1}$, ${\overline{A}}_{2} \dashv {\bar{R}}_{2}$, and ${\overline{A}}_{3} \dashv {\bar{R}}_{3}$) and concentration decay ${\mu }_{miR-21}{\overline{R}}_{1}$, ${\mu }_{miR-142}{\overline{R}}_{2}$, and ${\mu }_{miR-10{\overline{R}}_{3}}$. The second terms in (1)–(3) denote the autocatalytic activity with the inhibition process, with parameters for the autocatalytic production rates *τ*_1_–*τ*_3_ and Hill-type inhibition saturation constants *ϕ*_1_–*ϕ*_3_. In similar fashion, autocatalytic activities with the inhibition of TGF-*β* (${\overline{R}}_{1} \dashv \overline{T}$, ${\overline{R}}_{2} \dashv \overline{T}$, and ${\overline{R}}_{3} \dashv \overline{T}$) and SMAD (${\overline{R}}_{1} \dashv \overline{S}$, ${\overline{R}}_{2} \dashv \overline{S}$, and ${\overline{R}}_{3} \dashv \overline{S}$) in (4) and (5) still observe the same fractional form for their inhibition terms, with parameters for the autocatalytic production rates *τ*_4_ and *τ*_5_ and Hill-type constants *ϕ*_4_ and *ϕ*_5_ wherein the inhibition processes by the seizure-modifying miRNAs are expressed as functions *G*_4_ and *G*_5_ with respect to the miRNA concentrations ${\overline{R}}_{1}$, ${\overline{R}}_{2}$, and ${\overline{R}}_{3}$. The rate changes for TGF-*β* and SMAD concentrations are also regulated by the signaling sources at fixed rates *f*_TGF-*β*_, and *f*_SMAD_ and concentration decay ${\mu }_{{\rm{T}}{\rm{G}}{\rm{F}}-\beta }\bar{T}$, and ${\mu }_{SMAD}\overline{S}$, with SMAD activated by TGF-*β* ($\overline{T}\to \overline{S}$) at rate *λ*_TGF-*β*_ as its downstream signaling pathway.

In (1)–(3), high antagomir concentrations ${\overline{A}}_{1}$, ${\overline{A}}_{{2}}$, and ${\overline{A}}_{3}$ inhibit their targeted miRNAs ${\overline{R}}_{1}$, ${\overline{R}}_{2}$, and ${\overline{R}}_{3}$ through the positive functions ${G}_{1}({\overline{A}}_{1})$, ${G}_{2}({\overline{A}}_{2})$, and ${G}_{3}({\overline{A}}_{3})$, respectively. The mathematical conditions are then given as

9 $$\partial {G}_{1}/\partial {\bar{A}}_{1} > 0,\,\partial {G}_{2}/\partial {\bar{A}}_{2} > 0,\,\,\,\,\,\,\,\,\,\,\,\partial {G}_{3}/\partial {\overline{A}}_{3} > 0,$$

$\forall {\overline{A}}_{1},{\overline{A}}_{2},{\overline{A}}_{3}\ge 0$. The miRNA-mediated suppression of TGF-*β* and SMAD described in (4) and (5) is represented by the functions ${G}_{4}({\overline{R}}_{1},{\overline{R}}_{2},{\overline{R}}_{3})$ and ${G}_{5}({\overline{R}}_{1},{\overline{R}}_{2},{\overline{R}}_{3})$, respectively. Similarly, the conditions

10 $$\begin{array}{rcl}\partial {G}_{4}/\partial {\bar{R}}_{1} & > & 0,\,\partial {G}_{4}/\partial {\bar{R}}_{2} > 0,\,\,\,\,\,\,\,\,\,\partial {G}_{4}/\partial {\bar{R}}_{3} > 0,\\ \partial {G}_{5}/\partial {\bar{R}}_{1} & > & 0,\,\partial {G}_{5}/\partial {\bar{R}}_{2} > 0,\,\,\,\,\,\,\,\,\,\partial {G}_{5}/\partial {\bar{R}}_{3} > 0,\end{array}$$

$\forall {\overline{R}}_{1},{\overline{R}}_{2},{\overline{R}}_{3}\ge 0$ are also necessary. The functions representing the inhibition processes can then be expressed as

11 $$\begin{array}{lcl}{G}_{1}({\overline{A}}_{1}) & = & {{\overline{A}}_{1}}^{2},\,{G}_{2}({\overline{A}}_{2})={{\overline{A}}_{2}}^{2},\,{G}_{3}({\overline{A}}_{3})={{\overline{A}}_{3}}^{2},\\ {G}_{4}({\bar{R}}_{1},{\bar{R}}_{2},{\bar{R}}_{3}) & = & {{\bar{R}}_{1}}^{2}+{{\bar{R}}_{2}}^{2}+{{\bar{R}}_{3}}^{2},\\ {G}_{5}({\bar{R}}_{1},{\bar{R}}_{2},{\bar{R}}_{3}) & = & {{\bar{R}}_{1}}^{2}+{{\bar{R}}_{2}}^{2}+{{\bar{R}}_{3}}^{2}.\end{array}$$

Non-dimensionalising the parameters and variables then gives us

12 $$\begin{array}{lll}{A}_{1} & = & \frac{{\overline{A}}_{1}}{{A}_{1}^{* }},{A}_{2}=\frac{{\overline{A}}_{2}}{{A}_{2}^{* }},{A}_{3}=\frac{{\overline{A}}_{3}}{{A}_{3}^{* }},T=\frac{\bar{T}}{{T}^{* }},\\ {R}_{1} & = & \frac{{\bar{R}}_{1}}{{R}_{1}^{* }},{R}_{2}=\frac{{\bar{R}}_{2}}{{R}_{2}^{* }},{R}_{3}=\frac{{\bar{R}}_{3}}{{R}_{3}^{* }},S=\frac{\bar{S}}{{S}^{* }},\\ {\lambda }_{{R}_{1}} & = & \frac{{f}_{{\rm{m}}{\rm{i}}{\rm{R}}-21}}{{R}_{1}^{* }},{k}_{1}=\frac{{\tau }_{1}}{{R}_{1}^{* }},{k}_{2}={\phi }_{1},\alpha ={\upsilon }_{1}{({A}_{1}^{* })}^{2},\\ {\lambda }_{{R}_{2}} & = & \frac{{f}_{{\rm{m}}{\rm{i}}{\rm{R}}-142}}{{R}_{2}^{* }},{k}_{3}=\frac{{\tau }_{2}}{{R}_{2}^{* }},{k}_{4}={\phi }_{2},\beta ={\upsilon }_{2}{({A}_{2}^{* })}^{2},\\ {\lambda }_{{R}_{3}} & = & \frac{{f}_{{\rm{m}}{\rm{i}}{\rm{R}}-10}}{{R}_{3}^{* }},{k}_{5}=\frac{{\tau }_{3}}{{R}_{3}^{* }},{k}_{6}={\phi }_{3},\gamma ={\upsilon }_{3}{({A}_{3}^{* })}^{2},\\ {\lambda }_{T} & = & \frac{{f}_{{\rm{T}}{\rm{G}}{\rm{F}}-\beta }}{{T}^{* }},{k}_{7}=\frac{{\tau }_{4}}{{T}^{* }},{k}_{8}={\phi }_{4},\\ \delta & = & {\upsilon }_{4}{({R}_{1}^{* })}^{2},\epsilon ={\upsilon }_{4}{({R}_{2}^{* })}^{2},\zeta ={\upsilon }_{4}{({R}_{3}^{* })}^{2},\\ {\lambda }_{S} & = & \frac{{f}_{{\rm{S}}{\rm{M}}{\rm{A}}{\rm{D}}}}{{S}^{* }},{k}_{9}=\frac{{\tau }_{5}}{{S}^{* }},{k}_{10}={\phi }_{5},\lambda ={\lambda }_{{\rm{T}}{\rm{G}}{\rm{F}}-\beta },\\ \eta & = & {\upsilon }_{5}{({R}_{1}^{* })}^{2},\theta ={\upsilon }_{5}{({R}_{2}^{* })}^{2},\kappa ={\upsilon }_{5}{({R}_{3}^{* })}^{2},\\ {\mu }_{{A}_{1}} & = & {\mu }_{{\rm{a}}{\rm{n}}{\rm{t}}{\rm{i}}-{\rm{m}}{\rm{i}}{\rm{R}}-21},{\mu }_{{A}_{2}}={\mu }_{{\rm{a}}{\rm{n}}{\rm{t}}{\rm{i}}-{\rm{m}}{\rm{i}}{\rm{R}}-142},\\ {\mu }_{{A}_{3}} & = & {\mu }_{{\rm{a}}{\rm{n}}{\rm{t}}{\rm{i}}-{\rm{m}}{\rm{i}}{\rm{R}}-10},{\mu }_{{R}_{1}}={\mu }_{{\rm{m}}{\rm{i}}{\rm{R}}-21},{\mu }_{{R}_{2}}={\mu }_{{\rm{m}}{\rm{i}}{\rm{R}}-142},\\ {\mu }_{{R}_{3}} & = & {\mu }_{{\rm{m}}{\rm{i}}{\rm{R}}-10},{\mu }_{T}={\mu }_{{\rm{T}}{\rm{G}}{\rm{F}}-\beta },{\mu }_{S}={\mu }_{{\rm{S}}{\rm{M}}{\rm{A}}{\rm{D}}},t=\bar{t}.\end{array}$$

Finally, the dynamical system for seizure regulation as illustrated in Fig. 7b thereby has the governing equations written in their dimensionless forms

13 $$\frac{{\mathrm{d}}{A}_{1}}{{\mathrm{d}}t}=-{\mu }_{{A}_{1}}{A}_{1},$$

14 $$\frac{{\mathrm{d}}{A}_{2}}{{\mathrm{d}}t}=-{\mu }_{{A}_{2}}{A}_{2},$$

15 $$\frac{{\mathrm{d}}{A}_{3}}{{\mathrm{d}}t}=-{\mu }_{{A}_{3}}{A}_{3},$$

16 $$\frac{{\mathrm{d}}{R}_{1}}{{\mathrm{d}}t}={\lambda }_{{R}_{1}}+\frac{{k}_{1}{k}_{2}^{2}}{{k}_{2}^{2}+\alpha {A}_{1}^{2}}-{\mu }_{{R}_{1}}{R}_{1},$$

17 $$\frac{{\mathrm{d}}{R}_{2}}{{\mathrm{d}}t}={\lambda }_{{R}_{2}}+\frac{{k}_{3}{k}_{4}^{2}}{{k}_{4}^{2}+\beta {A}_{2}^{2}}-{\mu }_{{R}_{2}}{R}_{2},$$

18 $$\frac{{\mathrm{d}}{R}_{3}}{{\mathrm{d}}t}={\lambda }_{{R}_{3}}+\frac{{k}_{5}{k}_{6}^{2}}{{k}_{6}^{2}+\gamma {A}_{3}^{2}}-{\mu }_{{R}_{3}}{R}_{3},$$

19 $$\frac{{\mathrm{d}}T}{{\mathrm{d}}t}={\lambda }_{T}+\frac{{k}_{7}{k}_{8}^{2}}{{k}_{8}^{2}+\delta {R}_{1}^{2}+\epsilon {R}_{2}^{2}+\zeta {R}_{3}^{2}}-{\mu }_{T}T,$$

20 $$\frac{{\mathrm{d}}S}{{\mathrm{d}}t}={\lambda }_{S}+\lambda T+\frac{{k}_{9}{k}_{10}^{2}}{{k}_{10}^{2}+\eta {R}_{1}^{2}+\theta {R}_{2}^{2}+\kappa {R}_{3}^{2}}-{\mu }_{S}S.$$

Consistent with the results from Venø et al., the regulatory model incorporated the negative feedback of antagomir-mediated signal networks to repress the seizure-modifying miRNA expressions^37^. Also included are the negative feedback of miRNA-mediated signal networks to inhibit the levels of the downstream signaling of TGF-*β* and SMAD. Note that the down-regulated *T* and *S* levels are indications that seizures induced by Kainic Acid occurred, leading to the *seizure state*. On the other hand, seizure occurrence is reduced when *T* and *S* levels are upregulated *anti-seizure state*. Hence, the modeling framework characterizes the phenotypic switch between the two states when *T* and *S* levels cross their respective thresholds, denoted as *th*_*T*_ and *th*_*S*_. The threshold values were chosen based on the TGF-*β* and SMAD concentration trajectories of the dynamical system as described in (13)–(20), which are measured in FC over control.

### Characterization thresholds

The baseline antagomir levels are set to *A*_1_ = *A*_2_ = *A*_3_ = 1.0 FC over control, while the minimum antagomir levels are set to *A*_1_ = *A*_2_ = *A*_3_ = 0.0 FC over control, which are consistent with the units used for the molecular concentrations. The phenotypic thresholds *th*_*T*_ for TGF-*β* and *th*_*S*_ for SMAD define the anti-seizure $({{\mathbb{X}}}_{a})$ and seizure $({{\mathbb{X}}}_{s})$ states mathematically as

21 $${{\mathbb{X}}}_{a}=\{(T,S)\in {{\mathbb{R}}}^{2}:T > t{h}_{T},S > t{h}_{S}\},$$

and

22 $${{\mathbb{X}}}_{s}=\{(T,S)\in {{\mathbb{R}}}^{2}:T < t{h}_{T},S < t{h}_{S}\}.$$

### Experimental data and parameter estimation

**Fig. 9:**
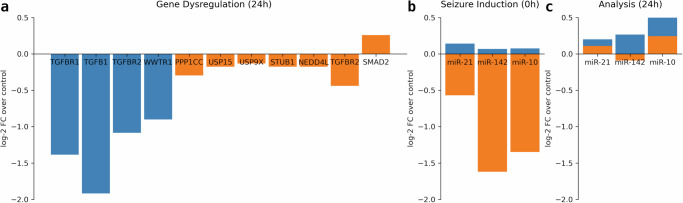
Dysregulated genes and miRNAs at different stages in the experimental setup. **a** Bar charts for the significantly down-regulated mRNAs (blue bars) and proteins (orange bars) associated with TGF-*β* signaling when the hippocampus slice was analyzed 24 h after SE induction. **b** Dysregulation of miR-21a-5p, miR-142a-5p, and miR-10a-5p at SE induction by an intraamygdala microinjection of Kainic Acid under both scrambled control (blue bars) and antagomir pre-injection (orange bars) treatments measured in ${\log }_{2}$(FC over control). **c** Dysregulation of miR-21a-5p, miR-142a-5p, and miR-10a-5p during Western blot analysis of the brain slice for scrambled controls (blue bars) and antagomir pre-injections (orange bars) in ${\log }_{2}$(FC over control).

Since the formulated dimensionless mathematical model contained unknown parameter values, they were estimated based on existing empirical data provided in the supporting information in Venø et al. and fitted to the experimental observations based on the structure of the model^37^. Parameter estimation for the mathematical model involves the quantification of gene dysregulation shown in Fig. 9a, with the statistically significant downregulated mRNAs (blue bars) and proteins (orange bars) associated with TGF-*β* signaling measured at 24 h after SE induction during which Western blot analysis was performed on the hippocampus slice, as demonstrated in Venø et al.^37^. The lower relative expressions of the three seizure-modifying miRNAs at the time of SE induction under antagomir pre-injections (orange bars) are shown in Fig. 9b, as compared to those with higher relative expressions under scrambled controls (blue bars), thereby affirming its anti-seizure effects. miRNA levels during Western blot analysis of the hippocampus slice under both treatments are also illustrated in Fig. 9c. These essentially provide a basis for the seizure suppression strategies proposed in this study. Furthermore, the parameter values denoting the source terms in Supplementary Table 1, decay terms in Supplementary Table 2, and inhibition terms in Supplementary Table 3 were used to evaluate the model equations in (13)–(20).

## Supplementary information

Supplementary Information

## Data Availability

The datasets analyzed in the current study are available as Supporting Information Datasets S01–S04 in 10.1073/pnas.1919313117.
