# Supplementary material for: Modeling the microRNA regulation of TGF-β/SMAD signaling pathways for seizure control in temporal lobe epilepsy
Source: NPJ Syst Biol Appl. 2026 Jan 15;12:21. doi: 10.1038/s41540-025-00643-6 (PMC12881450; doi:10.1038/s41540-025-00643-6)
Supplement: Supplementary file 1 — Supplementary Information [file 41540_2025_643_MOESM1_ESM.pdf]

Modeling the microRNA Regulation of TGF-β/SMAD Signaling Pathways for Seizure Control in Temporal Lobe Epilepsy

Kurt J.A. Pumares*[1], Daniel P. Martins[2], Aiman Khalil[1], Jochen H.M. Prehn[3], and Deirdre Kilbane[1]

Supplementary Table I: Source Parameters. Estimated values of the signaling source and activation rate parameters of all three epilepsy-associated microRNAs and the downstream TGF-β/SMAD signaling pathways used in fitting the model equations.

Parameter	Value	Description
λ_{R_1}	0.5 min^{-1}	Signaling source of miR-21a-5p
λ_{R_2}	0.5 min^{-1}	Signaling source of miR-142a-5p
λ_{R_3}	0.5 min^{-1}	Signaling source of miR-10a-5p
λ_T	1.0 min^{-1}	Signaling source of TGF-β
λ_S	1.0 min^{-1}	Signaling source of SMAD2/3:4
λ	0.5 min^{-1}	Activation rate of SMAD2/3:4 by TGF-β

Supplementary Table II: Decay Parameters. Estimated values of the relative decay rate parameters of the three antagomirs and their target epilepsy-associated microRNAs, as well as the downstream TGF-β/SMAD signaling pathways also used in fitting the model equations.

Parameter	Value	Description
μ_{R_1}	1.81 min^{-1}	Relative decay rate of miR-21a-5p
μ_{R_2}	1.91 min^{-1}	Relative decay rate of miR-142a-5p
μ_{R_3}	1.9 min^{-1}	Relative decay rate of miR-10a-5p
μ_T	1.1 min^{-1}	Relative decay rate of TGF-β
μ_S	1.7 min^{-1}	Relative decay rate of SMAD2/3:4
μ_{A_1}	0.1 min^{-1}	Relative decay rate of anti-miR-21a-5p
μ_{A_2}	0.1 min^{-1}	Relative decay rate of anti-miR-142a-5p
μ_{A_3}	0.1 min^{-1}	Relative decay rate of anti-miR-10a-5p

Supplementary Table III: Inhibition Parameters. Estimated values of the autocatalytic production rate, Hill-type coefficient, and inhibition strength parameters of all three epilepsy-associated microRNAs and the downstream TGF-β/SMAD signaling pathways used in fitting the model equations.

Parameter	Value	Description
k_1	1.0 min^{-1}	autocatalytic production rate of miR-21a-5p
k_2	0.1 min^{-1}	miR-21a-5p Hill-type coefficient
α	4.0 min^{-1}	Inhibition strength of miR-21a-5p by anti-miR-21a-5p
k_3	1.0 min^{-1}	autocatalytic production rate of miR-142a-5p
k_4	0.01 min^{-1}	miR-142a-5p Hill-type coefficient
β	4.0 min^{-1}	Inhibition strength of miR-142a-5p by anti-miR-142a-5p
k_5	1.0 min^{-1}	autocatalytic production rate of miR-10a-5p
k_6	0.07 min^{-1}	miR-10a-5p Hill-type coefficient
γ	4.0 min^{-1}	Inhibition strength of miR-10a-5p by anti-miR-10a-5p
k_7	1.0 min^{-1}	autocatalytic production rate of TGF-β
k_8	1.0 min^{-1}	TGF-β Hill-type coefficient
δ	1.0 min^{-1}	Inhibition strength of TGF-β by miR-21a-5p
ϵ	1.0 min^{-1}	Inhibition strength of TGF-β by miR-142a-5p
ζ	1.0 min^{-1}	Inhibition strength of TGF-β by miR-10a-5p
k_9	1.0 min^{-1}	SMAD2/3:4 autocatalytic production rate
k_{10}	1.0 min^{-1}	SMAD2/3:4 Hill-type coefficient
η	1.0 min^{-1}	Inhibition strength of SMAD2/3:4 by miR-21a-5p
θ	1.0 min^{-1}	Inhibition strength of SMAD2/3:4 by miR-142a-5p
κ	1.0 min^{-1}	Inhibition strength of SMAD2/3:4 by miR-10a-5p

[1] Walton Institute, South East Technological University, Waterford, Republic of Ireland e-mail: (see kurt.pumares@waltoninstitute.ie, aiman.khalil@waltoninstitute.ie, and deirdre.kilbane@waltoninstitute.ie)

[2] School of Computer Science and Electronic Engineering, University of Essex, Colchester, United Kingdom e-mail: (see daniel.martins@essex.ac.uk)

[3] Department of Physiology and Medical Physics, Royal College of Surgeons in Ireland, Dublin, Republic of Ireland e-mail: (see jprehn@rcsi.ie)

* Corresponding Author e-mail: (see kurt.pumares@waltoninstitute.ie)
